# Barriers to clinical adoption of pharmacogenomic testing in psychiatry: a critical analysis

**DOI:** 10.1038/s41398-021-01600-7

**Published:** 2021-10-06

**Authors:** Catherine R. Virelli, Ayeshah G. Mohiuddin, James L. Kennedy

**Affiliations:** 1 Tanenbaum Centre for Pharmacogenetics, Campbell Family Mental Health Research Institute, Centre for Addiction and Mental Health, Toronto, ON Canada; 2 Translational Research Program, Institute of Medical Science, University of Toronto, Toronto, ON Canada; 3 Department of Psychiatry, University of Toronto, Toronto, ON Canada

**Keywords:** Scientific community, Pharmacogenomics, Personalized medicine

## Abstract

Pharmacogenomics (PGx) is the study of genetic influences on an individual’s response to medications. Improvements in the quality and quantity of PGx research over the past two decades have enabled the establishment of commercial markets for PGx tests. Nevertheless, PGx testing has yet to be adopted as a routine practice in clinical care. Accordingly, policy regulating the commercialization and reimbursement of PGx testing is in its infancy. Several papers have been published on the topic of challenges, or ‘barriers’ to clinical adoption of this healthcare innovation. However, many do not include recent evidence from randomized controlled trials, economic utility studies, and qualitative assessments of stakeholder opinions. The present paper revisits the most cited barriers to adoption of PGx testing: evidence for clinical utility, evidence for economic effectiveness, and stakeholder awareness. We consider these barriers in the context of reviewing PGx literature published over the past two decades and emphasize data from commercial PGx testing companies, since they have published the largest datasets. We conclude with a discussion of existing limitations to PGx testing and recommendations for progress.

## Introduction

Pharmacogenomics (PGx) is a field of research primarily concerned with genetic influences on an individual’s ability to metabolize medications. In drug metabolism, genetic variants lead to altered function of liver enzymes that, in turn, creates variation in blood levels and types of metabolites across clients. Certain groups of genetic variants are associated with a metabolizer phenotype. These phenotypes describe the way in which an individual is most likely to respond to medications metabolized by the variant-containing gene. Historically, four metabolizer phenotypes have been identified: poor metabolizer (PM); intermediate metabolizer (IM); extensive metabolizer (EM); and rapid metabolizer (RM), or ultrarapid metabolizer (UM). EM refers to wildtype individuals i.e., those with normal metabolic activity. Clinical dosing standards are generally developed under the assumption of an EM phenotype for a given gene. PM and UM phenotypes are considered the most clinically relevant, as they are associated with adverse response or lack of response to medication, respectively [1–3]. Important exceptions for PM’s are in the case of medications that are prodrugs and need to be converted to their active form by the liver enzyme. For example, codeine is a prodrug converted to its active metabolite, morphine by the hepatic *CYP2D6* enzyme to provide its analgesic effect. *CYP2D6* gene PMs may experience insufficient pain relief due to lower morphine levels and *CYP2D6* gene UMs may experience symptoms of morphine toxicity due to more rapid conversion of codeine [4]. Individuals with a PM phenotype inherit two alleles with near-absent or absent function. Consequently, they are slow or unable to metabolize certain medications at the standard dose, leading to higher blood levels of these medications and, accordingly, increased potential for side effects. Conversely, individuals with a UM phenotype metabolize medications too quickly and blood levels are too low for therapeutic effect. Clients with this phenotype will, therefore, require higher than standard doses to experience a clinical effect [1–3]. Thus, a key benefit of PGx testing is the identification of medications to which a client is least (and most) likely to suffer side effects. To date, research in this area has enabled the examination of thousands of variants, leading to big data approaches that hold promise for many sophisticated healthcare applications in the future.

Pharmacodynamic (PD) targets have also been used in the development of PGx testing though there is less published on PD gene testing than pharmacokinetic testing. DRD2 dopamine receptor, COMT, and SLC6A4 serotonin transporter are genes frequently implicated in neuropsychiatric medication action and inclusion of such targets in PGx testing may help to increase treatment efficacy and limit undesired effects of medications [5].

PGx testing has particular relevance to psychiatric practice. Medication is the standard of care for most debilitating psychiatric illnesses. However, many medications commonly used to treat mental illnesses (i.e., psychotropic medications) have substantial adverse drug reaction and side effect profiles [6, 7]. Thus, clients seeking psychiatric treatment can be particularly susceptible to undesired side effects or adverse events [8, 9]. Unfortunately, such experiences are common, due in large part to the ongoing ‘trial-and-error’ approach to medication prescription. According to this method, prescription choices are guided, initially, by various standards of care and, subsequently, by observations of the client’s response to the medication. This method puts the client at increased risk of suffering adverse drug reactions and side effects. The fact that most psychotropic medications take four to six weeks to show an effect exacerbates this risk [10]. As trial-and-error frequently involves prescription changes, it also leads to considerable healthcare expenditures.

PGx testing has potential to mitigate concerns associated with trial-and-error. First, the client’s metabolizer phenotype is identified, based on their sampled DNA. Subsequent analysis depends on the type of test being used. Tests that assess metabolizer status based on a single gene use gene-drug pair information [from Clinical Pharmacogenetics Implementation Consortium guidelines (CPIC), for example] to predict the response a client will have to one or more commonly prescribed medications metabolized by the targeted gene [11]. Multi-gene tests assess metabolizer status based on variants from multiple genes. These tests often use proprietary algorithms to produce a list of commonly prescribed medications to which an individual is more likely to have beneficial outcomes, as well as those to which they are most likely to endure side effects. These algorithms may be developed using similar guidelines used for single-gene tests. However, not all multi-gene tests use proprietary algorithms, for example non-proprietary CPIC guidelines recommend measuring both CYP2D6 and CYP2C19 in order to determine optimal dosing for tricyclic antidepressants. The ability to target multiple genes at once confers different clinical and economic outcomes, relative to single-gene tests. In this paper, we focus our discussion on these multi-gene tests.

Several proprietary PGx tests, with varying levels of evidence for improvement of outcomes, are currently available to consumers [12]. However, this innovation has yet to be formally adopted in most healthcare institutions, internationally.

Issues preventing clinical adoption of PGx, commonly referred to as ‘barriers’ to clinical adoption, are often shared by industry and healthcare systems worldwide. Important developments have occurred recently in addressing these barriers. Acknowledging this, the authors set out to conduct a comprehensive review of commonly identified barriers to clinical adoption, with our discussion focusing on the most current and relevant evidence.

## Methods

The authors conducted a comprehensive review of the literature using PubMed, Cochrane, Scopus, and OVID databases. Search terms used in each phase of the literature search are provided in Supplementary Table 1. Subsequently, the authors investigated further each of the commonly cited barriers across the identified papers. Additional relevant studies were identified via references from these studies. Inclusion criteria common to all phases of the review included articles peer-reviewed, and full-text articles available in English. The results of our review were in some cases simplified due to the large number of studies.

### Phase One: Barriers to clinical adoption of PGx testing

In addition to meeting the common inclusion criteria, articles considered in this initial phase of the literature search addressed explicitly the subject of clinical adoption of PGx testing (i.e., regarding either the process of PGx implementation or barriers to this process). Inclusion priority was given to studies published between 2015 and 2020. This phase of the review largely identified review papers discussing the topic of barriers to adoption.

From this phase, several common barriers were identified. They included: evidence for the clinical utility of PGx testing, evidence for the cost-effectiveness of PGx testing, and physician knowledge of PGx testing. Subsequently, the authors conducted a second review of the literature to investigate further each of these barriers.

### Phase Two: Commonly cited barriers

For the ‘clinical utility’ barrier, papers were excluded from consideration if the methods and/or test used had no relevance to psychiatry, if the study sample included individuals under the age of 18, or if the study in question was a case study or otherwise had a relatively small sample size (*n* ≤ 150). In addition, inclusion priority was given to randomized controlled trials (RCTs).

Unique inclusion criteria for the ‘cost-effectiveness’ barrier included cost-utility or cost-effectiveness analyses that specified the PGx test used. Cost-utility analyses determine the number of Quality Adjusted Life Years (QUALYs) conferred to clients by a target intervention, relative to treatment as usual (TAU). Cost-effectiveness analyses assess the health costs of the intervention as they relate to a target health outcome [i.e., scores on clinical surveys, hospitalization records, etc.; see Verbelen et al. for further explanation of these terms] [13].

For the ‘physician education’ barrier, inclusion priority was given to studies that solicited opinions directly from physicians regarding their experience with and/or perspectives of PGx testing.

## Results

**Fig. 1:**
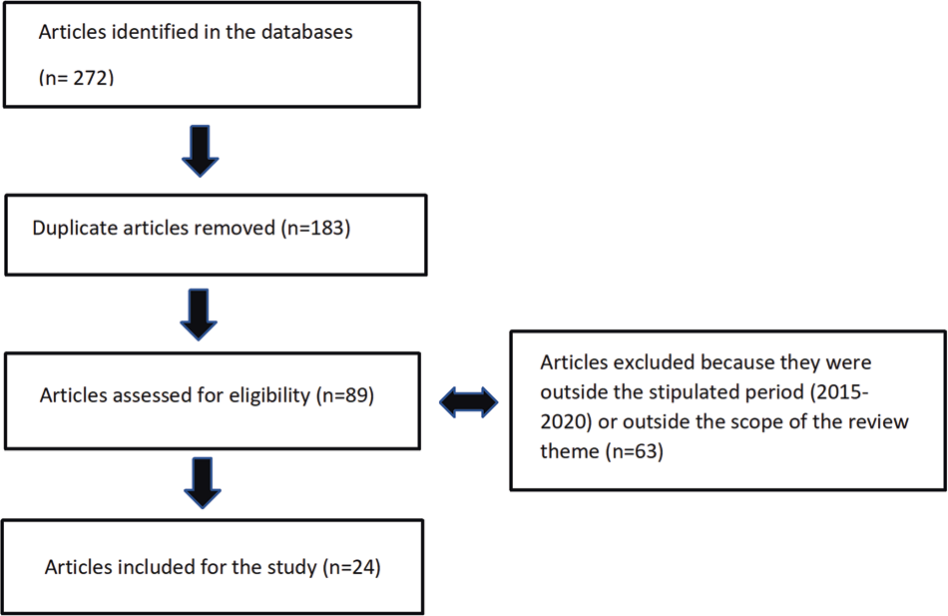
Flow chart illustrating the second phase of the literature review.

A total of 46 studies matching inclusion criteria were identified in the first phase of the literature search (i.e., discussing overall barriers to adoption of PGx testing in various geographic areas). Of all barriers identified in this phase, three were cited commonly across studies: (i) evidence for the clinical utility of PGx testing; (ii) evidence for the cost-effectiveness of PGx testing; and (iii) physician education regarding PGx testing (including PGx research, generally, as well as procedures associated with testing). These barriers were investigated in the second phase of the literature search (see supplementary material - Table 2). After screening article summaries for relevance, the final search outcome yielded 3 articles related to clinical utility of PGx testing (five included); 33 articles related to cost-effectiveness of PGx testing (13 included); and 21 articles related to physician education of PGx testing (six included) [Fig. 1].

In the next section, we provide more detailed results, and critical analysis, from the second phase of the literature search.

### Barrier 1: Clinical utility (and efficacy)

The overall paucity of high-quality, published evidence for the clinical efficacy and utility of PGx testing is a prominent issue preventing clinical adoption [14, 15]. A major underlying factor hindering evidence of clinical efficacy is the general lack of published randomized controlled trials (RCTs) on this subject; furthermore, the potential impact of the relatively few published RCTs has been a subject of controversy. Following is a discussion of evidence from available RCTs, as well as challenges to their generalizability.

### Randomized clinical trials (RCTs)

Several clinical trials have been published showing significant outcomes in favor of clinical utility [16–18]. As clinical utility is highly dependent on the PGx test being assessed, we discuss findings according to the test used in each study.

### GeneSight Psychotropic (Myriad Genetics)

An RCT assessing clinical utility of the GeneSight Psychotropic test included 1398 US clients diagnosed with major depressive disorder (MDD) with 717 patients receiving treatment as usual (TAU) and 681 patients with guided-treatment [16]. Results showed significant clinical improvements when physicians used GeneSight PGx test results to switch to a medication categorized as congruent by test guidance, versus those who remained on the guidance-incongruent medication (*p* = 0.002). Of note the primary outcome of symptom improvement was not statistically significant, rather it was improvements in response and remission rates of MDD that were found to be statistically significant [16]. This RCT showed greater overall improvements in remission (*p* = 0.007) and response [i.e., *a* > 50% decrease in scores on the Hamilton Depression Rating Scale (HAM-D) between baseline and week 8 follow-up, (*p* = 0.013) for participants whose physicians used PGx testing to guide their prescription decisions, compared to those who did not [i.e., TAU] [16].

### IDgenetix (formerly NeuroIDgenetix; Althea Dx)

The effects of PGx testing using the IDgenetix test have been assessed in one US clinical trial. The participant sample included 685 clients with a diagnosis of major depression, anxiety disorder (independent of depression), or major depression comorbid with anxiety disorder, respectively [17]. In this trial, clients with severe depression (i.e., HAMD score > 24; *n* = 40) whose physicians used the IDgenetix PGx test showed greater improvements in response (*p* = 0.001) and remission (*p* = 0.02) relative to TAU, per HAM-D scores, at 12-week follow-up. This clinical trial also showed significant reductions in anxiety symptoms, at all data collection points, for participants who received PGx testing relative to those in the control group [*p* (8 wks) = 0.02] [17].

### Neuropharmagen (AB-Biotics)

A double-blinded randomized clinical trial has been conducted for the Neuropharmagen Pharmacogenetic Test. This trial found significant improvements in HAM-D scores at six-week follow-up (*p* = 0.04) for participants who received PGx-guided treatment, though the significance of this improvement was lost at the 12-week follow-up (*p* = 0.08) [18]. In this trial, participants who had treatment guided by PGx testing also reported greater perceived response to treatment than those in the TAU group, as measured by the Patient Global Impression of Improvement (PGI-I) scale (47.8 % vs. 36.1 %; *p* = 0.05) [18].

### Meta-analyses of RCTs

To date, few studies have compared RCTs on PGx testing as a decision support tool. Bousman et al., presented a meta-analysis of five RCT’s where PGx-guided treatment (*n* = 887) was 1.71 (*p* = 0.005) times more likely to attain symptom remission compared to those who received TAU (*n* = 850) [19]. Similarly, Rosenblat et al. compared four RCTs where guided treatment (*n* = 352) was 1.74 (*p* = 0.02) times more likely to attain symptom remission compared to those who received TAU (*n* = 383) [20]. Meta-analyses represent the highest quality evaluation of RCTs. These two studies show very consistent benefit of 1.7 times more improvement in remission when treatment is guided by PGx testing. Thus, at this relatively early stage there is promising positive support for the clinical utility of pharmacogenomic guided treatment.

### Challenges to generalizability of clinical utility

Several challenges have been identified regarding the generalizability of available evidence for clinical utility of PGx testing [14, 20]. Foremost, the authorship of each RCT includes individuals with vested interest in the success of the PGx test being analyzed [14, 20]. This conflict of interest, however carefully managed, presents an inevitable caveat to interpretation of results. Unfortunately, this issue cannot be addressed easily. RCTs are extremely costly and surpass the budgetary constraints of many public or academic research groups. Industry partnerships are a relatively accessible means to support academic researchers in their effort to produce scientifically meaningful data. Ultimately, the information often translates into innovations that improve the quality of client care. Furthermore, there is a lack of standardization across tests in terms of specifying which genetic variants are being labeled as important. There needs to be improvement in providing more detailed documentation of exactly which variants are being used to provide treatment recommendations.

The fact that clinical symptoms of mental illness may be influenced by a variety of lifestyle and environmental factors, independent of psychotropic medications, is another issue preventing universal generalizability of evidence for PGx clinical utility [21, 22]. Environmental factors include family stress, history of childhood trauma, substance abuse, diet, and exercise, among others. Absent controlling for such factors, it is difficult to make direct associations between clinical outcomes (such as response and remission) and PGx testing. Thus, future RCTs should include methods of accounting and controlling for these lifestyle variables.

Finally, as the concept of clinical utility extends to ease of use and access for clients, it is important to consider the needs and perspectives of this stakeholder group. Regarding this issue, the relative lack of individuals of non-European ancestry in PGx research participant samples, compared to those of European ancestry, is a major and under-discussed barrier to generalizability of clinical utility [23–26]. One contributing factor to this issue is a mistrust for genetics research, and its associated institutions, held by many communities of non-European ancestry [23–26]. One root of this mistrust is the historical oppression of these communities by physicians and genetics researchers. Several efforts have been made toward progress in this area [23–26]. Following the lead of these researchers, a step forward will include consultation of community members of non-European ancestry, and groups representing them, to identify their unique concerns regarding participation in genetic studies as well as possible ways in which to ensure a subjective feeling of comfort and safety in research participation. Until this issue of representation is resolved, arguments for the universal clinical utility of PGx testing are compromised, inevitably.

### Barrier 2: Cost-effectiveness

Many, but not all, studies identify PGx testing as cost-effective or cost-saving relative to treatment-as-usual [13, 27]. Generalizability of cost-effectiveness literature is influenced by several factors, one of the most important being the test used in a given analysis. For this reason, we discuss the evidence in this section as it pertains to tests with the largest body of evidence.

### GeneSight Psychotropic (Myriad Genetics)

Several economic analyses have been conducted for the GeneSight Psychotropic test. In the most recent of these, base-case analysis showed a remission rate 1.53-times greater for clients whose treatment was guided by PGx, relative to TAU, and a projected, relative increase of 0.17 Quality Adjusted Life Years (2.02 months) for the PGx-guided group [28]. The PGx-guided strategy was also determined to be cost-saving relative to TAU, per the standards of the UK National Institute for Health and Care Excellence (NICE), as well as those commonly used in the US (Incremental Cost-Effectivenes Ration or ICER = -$10,971.60 USD/QALY) [13, 28–30].

Another recent analysis assessed psychiatric medication cost savings based on pharmacy claims data from an RCT of GeneSight by Winner et al. Claims data from that US study, including drug prescriptions and associated costs, were used as a model for application to the Canadian context using the Ontario Drug Benefits formulary [28]. Results of this study showed savings of $1061 CAD per member per year (PMPY) for clients whose physicians prescribed a psychiatric medication congruent with GeneSight test results (*p* < 0.0001) [28].

Finally, an early analysis conducted by Winner et al. compared pharmacy claims data, over the course of one year, between a group of clients whose treatment had been guided by the GeneSight test (*n* = 1662) and a propensity-matched control group (*n* = 10,880) [31]. In this study, the mean increase of medication costs PMPY increased by $1725.24 USD from the pre-test period to the study end in the TAU group [31]. This significantly exceeded the mean increase in the GeneSight-guided group ($689.62 USD PMPY, *p* < 0.0001) [31]. Furthermore, it was reported that participants in the GeneSight-guided group with a primary diagnosis of anxiety disorder (*n* = 328) and whose physicians selected a medication congruent with (i.e., matching) their PGx test results had the greatest and most significant cost-savings of any other diagnostic category in the PGx-guided group [$6874.69 USD total savings; $5666.72 (congruent) vs. $12,652.41 (incongruent), *F* = 19.75, *p* < 0.0001] [31]. Those in the PGx-guided group with a primary diagnosis of depression and prescribed a congruent medication also saved significantly on medication costs [$3579.81 total savings; $7,715.17 (congruent) vs. $11,294.98 (incongruent), *F* = 7.26, *p* < 0.0001] [31].

The 2015 study by Winner et al. was analyzed further by Brown et al., who focused specifically on outcomes from the PGx-guided group (*n* = 1662) [31, 32]. The authors compared total medication costs between clients whose psychiatrist, primary care provider, or OB/GYN prescribed PGx test-congruent medications and those whose healthcare provider prescribed an incongruent medication [32]. They found that congruent prescriptions led to significant cost-savings for clients of primary care providers (total savings = $3998 USD, *p* < .001), and overall cost savings were significant for test-guided participants being treated by a primary care provider ($3998; *p* = 0.001) or psychiatrist ($1308, *p* = 0.013), but not OBs/GYN (*p* = 0.14), for a CNS illness [32]. This difference in significance for OBs/GYNs may be a result of the relatively fewer prescriptions of psychotropic medications in these medical practices.

### IDgenetix (formerly NeuroIDgenetix; Althea Dx)

Groessl et al. developed an economic model of savings from PGx-guided treatment using the IDGenetix test [33]. Test cost and participant claims data were based on the IDGenetix RCT by Bradley et al. [17, 33]. Results showed that total direct medical costs over three years were lower for participants with moderate to severe depression whose clinicians had used the IDGenetix test to guide their treatment ($44,697 USD vs. $47,295, respectively; OR ~ 0.94) [33]. The model also showed that clients with severe depression (HAM-D score > 24) incurred $41,215 of total healthcare costs at three years, compared to $47,025 in the TAU group [33]. Significance values were not reported. Markov modeling also predicted an increase of 0.10 QALYs in the IDgenetix-guided group, relative to those in the TAU group [33]. The authors attributed part of this difference to a lower likelihood of death by suicide in the IDgenetix-guided group [33].

In another study of the economic effectiveness of the IDgenetix test, participants whose treatment was guided by PGx had lower total costs relative to those who received treatment as usual [$14,124 USD, IDgenetix (95% CI 10,703–17,630), vs. $14,659, TAU (95% CI 10,384-19,275)] [34]. This study adopted Greenberg and colleagues’ definition of ‘total cost’: specifically, the sum of direct healthcare costs associated with major depressive disorder (medical services, prescriptions, etc.); costs associated with depression that is not classified as MDD; absenteeism and disability; and non-mental health medical services and prescriptions [34, 35].

### Genecept (GenoMind)

The most recent economic analysis of the Genecept test was a propensity-score matched, retrospective, case-control design [36]. This study compared outcomes (count of emergency room visits, count of inpatient and outpatient visits, respectively, and change in number of psychotropic drug prescriptions) at six-months follow-up post-test [36]. The participants who had used the Genecept test were compared to a matched control sample drawn from insurance claims data of Aetna, a large commercial insurance company [36]. The experimental group data was drawn from the consumer registry of the patent-holding company. Results showed that those in the PGx-tested group had significantly fewer emergency room visits (*p* < 0.0001); overall inpatient hospitalizations (*p* < 0.0001); and outpatient visits (*p* = 0.0003) [36]. Based on this information, the authors estimated a $1229 decrease in overall, unadjusted medical costs for the PGx-tested group [36]. These costs do not include that of the Gencept test, itself.

Interestingly, Genecept testing was used in a multicenter randomized double-blinded study among outpatients with MDD. Guided treatment (*n* = 151) vs, TAU (*n* = 153) did not exhibit a significant difference at week 8 (*p* = 0.53) in the Hamilton Depression Rating Scale (SIGH-D-17). However, PGx-guided treatment was found to be linked with improved rates of remission [37].

An earlier economic assessment of the Genecept PGx test examined client claims data over a four-month period [38]. Clients were divided into two groups: one group included those whose physicians had ordered the Genecept test, and the other included clients whose treatment was not guided by genetic testing. Participants whose physicians had ordered the Genecept test showed an average increase in pharmacy costs of $886, relative to an increase of $222 for controls [38]. Baseline and replication analyses showed significantly greater improvements in medication adherence for participants in the Genecept-guided group at the end of the four-week period, relative to the initial assessment (*P* *=* 2.40 × 10^−4^) [38]. Thus, the increase in pharmacy costs may be attributed to Genecept-guided prescription changes. However, the study did not disclose rates of physicians’ congruence of their prescription decision with the test results. The TAU medications might be expected to be older, more established, and thus off-patent and lower priced, while the newer medications that the test might guide the physician to prescribe may be more expensive. As such, it is unclear whether the outcomes measured are a result of PGx test guidance.

The above discussion of evidence highlights some issues related to interpretation and generalizability of economic utility studies. These limitations to generalizability are important to understand, particularly with regard to clinical adoption.

### Challenges to generalizability of economic benefits

The evidence cited above for the cost-utility and/or cost-effectiveness of PGx testing must be interpreted cautiously. As is the case with generalizing clinical utility outcomes, the same, non-pharmacological factors that influence clinical improvement (stress, lifestyle changes, therapeutic intervention, etc.) may influence economic assessments, as their influence on outcome may not be sufficiently accounted for by researchers. Absent doing so, one cannot wholly attribute changes in overall healthcare costs, to PGx testing alone.

Additional issues with generalizability exist due to the lack of standardization of the following: measurement of PGx testing outcomes in economic effectiveness studies; methodology of these studies; and PGx testing results [15, 32]. Overcoming these standardization issues is important for progress in this field [39]. In addition, the costs of commercially available PGx tests are highly variable. This will influence, inevitably, cost-effectiveness, as well as clients’ ability to access the test. Collectively, these issues preclude an unequivocal classification of PGx testing as cost-effective.

Reimbursement -- i.e., complete, or partial coverage of test cost by an insurance provider or institution -- is another key factor influencing cost-effectiveness and cost-utility of PGx testing. This is a particularly complex issue. Government and private insurance entities generally desire robust cost-utility and cost-effectiveness evidence for a given product in order to proceed with reimbursement, and the limitations to the literature discussed in this section have, historically, prevented widespread progress on this front. On the other hand, partial or complete reimbursement through an insurer can be a key deciding factor for client access to and uptake of PGx testing, as well as physicians’ willingness to order these tests [40–42]. Reimbursement for PGx testing -- particularly by government health insurance plans, where available -- can also help reduce overall costs for clients and avoid inequities in test access between individuals of different socioeconomic classes. Thus, as policy continues to develop regarding use of PGx testing in healthcare contexts, governments ought to also consider a range of approaches to offsetting costs of this testing for their citizens.

A final note of caution regarding interpretation and translation of PGx cost-effectiveness literature is the importance of geographical context. Factors relevant to cost-effectiveness may vary by healthcare system. At the time of writing, the majority of cost-effectiveness studies have been conducted in and for clients in the United States. This presents obvious issues for other countries to interpret and implement results of these studies. Thus, stakeholders in countries that have generated fewer studies should develop economic models appropriate to their respective healthcare systems. They may do this by consulting medical research, gray literature, and other relevant economic models. For example, in the Canadian context, there are very few studies assessing cost-effectiveness of PGx testing with Canadian participants in Canadian healthcare institutions. However, outcomes from studies relevant to such an assessment may be used to populate economic models developed by researchers and/or government and private payors. For example, costs associated with major depression - including inpatient service utilization, hospital readmissions, medication costs, among others - have been assessed in at least three Canadian provinces [43–46]. Results from studies such as these can be used to develop PGx economic effectiveness models unique to the Canadian context. A similar process may be followed in other countries that lack these data for their specific client populations.

### Barrier 3: Physician education

Stakeholder groups relevant to implementation of PGx testing include, but are not limited to physicians, clients, pharmacists, medical trainees, insurance companies, and governments. There is very limited literature regarding these stakeholders and PGx. Thus, in this section, we focus on the areas with the most developed reporting, that is physician knowledge pertaining to PGx testing, as well as existing PGx guidelines that can serve as educational tools.

Lack of knowledge regarding PGx is a major barrier to clinical adoption and can be partially attributed to insufficient education on the subject. Indeed, in medical pedagogy, the quality of PGx instruction varies greatly across schools. Green et al. surveyed 90 department chairs from medical schools in the US and Canada to assess the extent of PGx education in their respective institutions [47]. A dedicated PGx course was available in only two medical schools. PGx education most often took place in second-year pharmacology courses, and 71.6% of respondents estimated that required teaching of curricular PGx content would take less than 4 h [47]. This disparity in PGx education has unfortunate effects on the confidence of physicians to use and interpret results from PGx tests. Haga et al. conducted a survey of 597 physicians board-certified in either family medicine or internal medicine, but not in a sub-specialty [48]. Just over half of respondents (51.4%; *n* = 306) felt inadequately informed about genetic testing; 22% (*n* = 131) stated that they had not previously received any form of PGx education; and 73% (*n* = 435) did not feel that their genetic training prepared them to use PGx tests or comprehend results [48].

The overall lack of education in PGx is particularly problematic because, as previously mentioned, testing is already available to clients through industry stakeholders. As physicians, and especially primary care providers, manage the health of many prospective PGx end users, they are among the most capable and important of all PGx stakeholders to engage critically in the implementation discourse, regardless of whether they choose to adopt testing in their clinic. This stated, even a relatively little amount of PGx education can have significant influences on physicians’ perceptions of clinical utility. At least three studies have shown strong associations between education in PGx, or self-reported knowledge thereof, and increased positive attitudes toward utility of PGx testing [42, 49, 50]. A 2015 survey of 168 psychiatrists and general practitioners with recent experience ordering pharmacogenomic tests found that 80% were satisfied or very satisfied with the genetic information provided by the tests, and 76% reported satisfactory or higher than satisfactory comprehension of test results [50]. Given that physicians who have experience ordering and administering the PGx test generally endorse being satisfied with the genetic information, it is possible that the act of reading the test instructions, and acting on the information provided, may provide significant educational benefit. Other assessments of physicians in non-psychiatric specialties support the association between knowledge of and/or experience with PGx testing and positive attitudes toward clinical utility [42, 49].

Here, we have limited our discussion to PGx education for physicians. However, there is a growing body of literature regarding integration of PGx testing and knowledge translation in pharmacy practice. We direct readers to this body of literature, as it makes a compelling case for the utility of engaging pharmacists in the implementation of PGx testing [51–56].

Of importance, PGx education is highly amenable to online delivery [57]. Thus, it is a feasible addition to medical school curricula and Continuing Medical Education programs, amid the ongoing COVID-19 pandemic.

## Discussion

The promise of PGx testing has been discussed in the scientific literature for nearly 70 years [58]. Strong interest from relevant healthcare stakeholders, including clients, has grown with the amount of evidence accumulated to date. However, PGx testing has yet to be widely adopted by public healthcare systems, internationally. As PGx testing is already available commercially, failure to adopt this testing in public healthcare institutions presents an inequality in access to a potentially treatment-altering healthcare innovation. Furthermore, as public healthcare institutions are subject to different confidentiality and conduct protocols than the private sector, this lack of adoption may also leave clients vulnerable to risks associated with less rigorous protocols.

With this in mind, here we have discussed several factors cited commonly in the scientific literature as major barriers to clinical adoption of PGx testing. Our hope is that this discussion of evidence, as well as limitations to its generalizability, may assist government and senior healthcare stakeholders with considerations for clinical adoption of PGx testing.

In the interest of maintaining a focused scope, we have not discussed ongoing efforts related to facilitating clinical adoption of PGx testing, making it more user-friendly, and expanding its applications. There is an ongoing body of work regarding the use of online interfaces (mobile apps, websites, hospital software, etc.) to display, discuss, and engage with PGx test results [59]. These interfaces, particularly those that integrate data from electronic health records, are essential to the sustainable implementation of PGx testing in clinics.

As discussed, overrepresentation of authors with vested interest in the PGx test being assessed is a limitation of research evaluating the clinical utility and cost-effectiveness of PGx testing. It should be noted that in general, new drugs and medical devices have their initial publications written by industry-academia partnerships. Nevertheless, the issue of vested interest remains, and ought to be addressed in a manner that preserves institutional capacity to conduct PGx research while also protecting the interests of physicians and clients. One way to accomplish this is by lobbying policymakers to develop regulations for these partnerships and improve upon existing ones. Such improvements could include protocols for maintaining user confidentiality, optimal data storage, and ensuring open access to RCT and economic assessment data, among others. Indeed, progress on this front has already begun. Several government institutions, internationally, have begun to develop policy regarding the safe collection of genetic data from clients. Examples of such policy include Bill S-201 (the Genetic Non-Discrimination Act) in Canada, the Genetic Information Non-Discrimination Act (GINA) and Health Insurance Portability and Accountability (HIPAA) in the United States, and the *Gendiagnostikgesetz* (Genetic Diagnosis Act) in Germany. Kalokairinou et al. review additional European legislation regulating direct-to-consumer PGx tests, specifically [60].

Policy regulating the development and use of pharmacogenomic testing is also important to ensure the equitable development and distribution of tests. As mentioned, the overrepresentation of individuals of European ancestry in PGx studies and associated genetic research is a major barrier to clinical adoption that has received limited attention in the scientific literature, relative to the barriers discussed in this review. Indeed, this issue extends to Genome-Wide Association Studies (GWAS) and other foundational methodologies from which researchers and companies draw genomic data. Specifically, most GWAS studies have been conducted using exclusively or near-exclusively individuals of European ancestry, and there are limitations to the generalizability of these data to populations of non-European ancestry [61–64]. Current PGx tests, which have been largely developed based on established function of drug-gene pairs in liver metabolism, still have limitations for clients of non-European ancestry. For example, clopigodrel, a commonly prescribed antiplatelet medication following myocardial infarction, is a prodrug primarily metabolized by *CYP2C19* (among other CYPP450 enzymes) into its active metabolite. It has been shown that African Americans with increased function of CYP2C19*17 allele are at an increased risk of bleeding events [65]. Further progress has been made on the issue regarding ancestry; for example, there is now extensive evidence of the association between the HLA-B-1502* variant and carbamazepine-induced Stevens-Johnson syndrome in individuals of East Asian descent [66]. Namely, Chinese, Malays, Thai, and Indian subgroups, but not in Japanese and Korean populations [67–69]. There is also some literature that identifies psychopharmacologically relevant variants for individuals of African ancestry [70, 71]. However, these data, as well as those for other non-European demographic groups, are limited relative to those for individuals of European ancestry. Addressing this issue will require an alternate research approach. Scientists must collaborate with leaders and organizations relevant to each of these non-European ancestry communities to develop safe and effective research engagement strategies. Scientific funding agencies should also prioritize community consultation studies to identify needs related to safe and responsible conduct of genetics research, and co-develop, with community members, protocols for research studies. Participatory action research is one technique that may be used to this end [72]. Researchers must also be accountable to the community and commit to meeting their stated needs. Such actions, while different from ordinary research practices, are necessary to responsibly engage individuals of non-European ancestry in genetics research. Furthermore, we argue, absent actions such as these, arguments for clinical utility and economic effectiveness of PGx testing are compromised.

This review has several limitations, one of which is that it was not registered with PROSPERO. Furthermore, as mentioned, this review focuses on the most discussed barriers to clinical adoption in the literature, based on results from the first phase of the literature search. A formal assessment of articles to identify frequently used terms was not conducted in this first phase of review. Thus, some may take issue with the scope of our selection. We encourage continued analysis of these and additional barriers (data privacy, access to lab facilities, etc.) in order to fully define the implementation landscape for clinical adoption. Nevertheless, as mentioned, it is also true that PGx testing has already been implemented in several jurisdictions across the globe, and companies operating independently of the healthcare sector have already begun marketing this test to consumers. Thus, with a view toward responsible adoption that protects the needs and interests of clients, we conclude this review with recommendations for stakeholders.

## Recommendations and conclusion

Healthcare stakeholders of authority in research and policymaking institutions should collaborate with local content experts, key opinion leaders, genetic counselors, genomics researchers, and community members of non-European ancestry to develop protocols for the safe and responsible engagement of individuals in these communities in PGx research. These stakeholders must be accountable to their collaborators and commit to meeting stated needs.

These stakeholders should also make considerable investments in developing community advisory boards (CABs) representative of local communities of non-European ancestry. CABs should be given authority to review and have full participation in the development of research protocols.

Policymaking stakeholders should mandate that genetic variants relevant to populations of non-European ancestry be included in commercial pharmacogenomic testing panels.

To facilitate policy development and reimbursement considerations by relevant stakeholders, research institutions should set guidelines for standardization of PGx research outcomes. These guidelines ought to include models for assessments of economic utility and effectiveness in PGx.

Regarding improved PGx education for physicians, academic and research institutions should collaborate and provide up-to-date instruction for medical students and residents. It is critical that this education include best practices for test result communication to clients.

Finally, government stakeholders should develop policy regulating healthcare-industry partnerships in PGx. Effective policy development may enable wider institutional and community participation in PGx research and discovery. It may also protect public interests via:

- Development of a code of conduct for stakeholders regarding the collection and handling of genetic data. This code may also set requirements for communication of test benefits and limitations, as well as receipt of informed consent.
- Assurance that genetic data is de-identified, stored securely, and subject to strict privacy regulations.

Overall, there appears to be great promise in the application of PGx testing in psychiatric care. Progress in addressing barriers to clinical adoption, identified in this review, should assist with actualizing the benefits of this innovation and advancing the potential of personalized medicine to improve healthcare systems worldwide.

## Supplementary information

Supplementary Material
