# Supplementary material for: Barriers to clinical adoption of pharmacogenomic testing in psychiatry: a critical analysis
Source: Transl Psychiatry. 2021 Oct 6;11:509. doi: 10.1038/s41398-021-01600-7 (PMC8492820; doi:10.1038/s41398-021-01600-7)
Supplement: Supplementary file 1 — Supplementary Material [file 41398_2021_1600_MOESM1_ESM.docx]

**Supplemental Material**

**Search Term Summary**

| **Search Phase** | **Search Terms** |
| --- | --- |
| **Phase One (General)** | Challenge AND Pharmacogenomic (1); Challenge AND pharmacogenomic testing (2) ; Barrier AND pharmacogenomic (3); Barrier AND pharmacogenomic testing (4); Barrier AND adoption AND pharmacogenomic (5); Challenge AND adoption AND pharmacogenomic (6); barriers AND genomic medicine (7); Clinical implementation of pharmacogenomic testing (8); Opportunities AND pharmacogenomic testing (9); (1)AND psychiatry; (2) AND psychiatry; (3) AND psychiatry; (4) AND psychiatry; (5) AND psychiatry; (6) AND psychiatry; (7) AND psychiatry; (8) AND psychiatry |
| **Phase Two (Clinical Utility)** | Clinical utility AND pharmacogenomic (1); Clinical utility AND pharmacogenomic (2); (1) AND psychiatry; (2) AND psychiatry |
| **Phase Two (Cost-effectiveness)** | cost-effectiveness AND pharmacogenomic (1); cost-utility AND pharmacogenomic (2); cost-effectiveness AND cost-utility AND pharmacogenomic (3); economic effectiveness AND pharmacogenomic (4); economic utility AND pharmacogenomic (5); (1) AND psychiatry; (2) AND psychiatry; (3) AND psychiatry; (4) AND psychiatry; (5) AND psychiatry |
| **Phase Two (Stakeholder Awareness)** | user awareness AND pharmacogenomic testing (1); user awareness AND pharmacogenetic testing (2); pharmacogenomics AND knowledge (3); pharmacogenetics AND knowledge (4); user awareness AND precision medicine (5); pharmacogenetics AND knowledge AND education (6); pharmacogenetics AND knowledge AND education AND training (7); (1)AND psychiatry; (2) AND psychiatry; (3) AND psychiatry; (4) AND psychiatry; (5) AND psychiatry; (6) AND psychiatry; (7) AND psychiatry |

Supplemental Table 1: Summary of terms used for literature search

**Summary of studies for each barrier**

| Barriers | Author | Title |
| --- | --- | --- |
| 1. Clinical utility and efficacy | Greden et al. (2019) | Impact of pharmacogenomics on clinical outcomes in major depressive disorder in the GUIDED trial: A large, patient- and rater-blinded, randomized controlled study. |
|  | Bradley, et al. (2018) | Improved efficacy with targeted pharmacogenetic-guided treatment of patients with depression and anxiety: A randomized clinical trial demonstrating clinical utility |
|  | Pérez, et al. (2017) | Efficacy of prospective pharmacogenetic testing in the treatment of major depressive disorder: Results of a randomized, double-blind clinical trial. |
|  | Bousman, et al. (2019) | Pharmacogenetic tests and depressive symptom remission: a meta-analysis of randomized controlled trials. Pharmacogenomics |
|  | Rosenblat, et al. (2018) | The effect of pharmacogenomic testing on response and remission rates in the acute treatment of major depressive disorder: A meta-analysis |
| 1. Cost-effectiveness | Berm, et al. (2016)  Verbelen, et al.  (2017)  Tanner, et al. (2020)  McCabe, et al. (2008)  Marseille, et al. (2015)  Tanner, et al. (2019)  Winner et al. (2015)  Brown et al. (2017)  Groessl et al.(2018)  Najafzadeh, et al. (2017)  Greenberg et al. (2015)  Perlis, et al. (2018)  Perlis, et al. (2020) | Economic evaluations of pharmacogenetic and pharmacogenomic screening tests: A systematic review. Second update of the literature.  Cost-effectiveness of pharmacogenetic-guided treatment: Are we there yet?  Cost-effectiveness of combinatorial pharmacogenomic testing for depression from the Canadian public payer perspective.  The NICE Cost Effectiveness Threshold: what it is and what that means.  Thresholds for the cost-effectiveness of interventions: Alternative approaches.  Canadian medication cost savings associated with combinatorial pharmacogenomic guidance for psychiatric medications.  Combinatorial pharmacogenomic guidance for psychiatric medications reduces overall pharmacy costs in a 1 year prospective evaluation  Economic utility: Combinatorial   pharmacogenomics and medication cost savings for mental health care in a primary care setting.  Cost-effectiveness of a pharmacogenetic test to guide treatment for major depressive disorder.    Economic evaluation of implementing a novel pharmacogenomic test (IDgenetix) to guide treatment of patients with depression or anxiety.  The economic burden of adults with major depressive disorder in the United States (2005 and 2010).  Pharmacogenetic testing among patients with mood an anxiety disorders is associated with decreased utilization and cost: A propensity-score matched study.  Randomized, controlled, participant‐ and rater‐ blind trial of pharmacogenomic test‐guided treatment versus treatment as usual for major depressive disorder |
| 1. Physician awareness | Stanek, et al. (2012) | Adoption of pharmacogenomic testing by US physicians: Results of a nationwide survey. |
|  | Green, et al. (2010) | Pharmacogenomics instruction in US and Canadian medical schools: Implications for personalized medicine. |
|  | Haga, et al. (2012) | Primary care physicians’ knowledge of and experience with pharmacogenetic testing. |
|  | Luzman, et al. (2016) | Physicians’ attitudes toward pharmacogenetic testing before and after pharmacogenetic education. |
|  | Walden, et al. (2015) | Physicians’ opinions following pharmacogenetic testing for psychotropic medication. |
|  | Ma, et al (2013) | A massive open online course on pharmacogenomics: Not just disruptive innovation but a possible solution. |

Supplemental Table 2: Summary of studies for each of the three barriers
